# Evolutionary Dynamics of MERS-CoV: Potential Recombination, Positive Selection and Transmission

**DOI:** 10.1038/srep25049

**Published:** 2016-05-04

**Authors:** Zhao Zhang, Libing Shen, Xun Gu

**Affiliations:** 1 State Key Laboratory of Genetic Engineering and MOE Key Laboratory of Contemporary Anthropology, School of Life Sciences, Fudan University, Shanghai, 200433, PR China; 2 Department of Genetics, Development, and Cell Biology, Iowa State University, Ames, IA, 50011, USA

## Abstract

Middle East respiratory syndrome coronavirus (MERS-CoV) belongs to beta group of coronavirus and was first discovered in 2012. MERS-CoV can infect multiple host species and cause severe diseases in human. We conducted a series of phylogenetic and bioinformatic analyses to study the evolution dynamics of MERS-CoV among different host species with genomic data. Our analyses show: 1) 28 potential recombinant sequences were detected and they can be classified into seven potential recombinant types; 2) The spike (S) protein of MERS-CoV was under strong positive selection when MERS-CoV transmitted from their natural host to human; 3) Six out of nine positive selection sites detected in spike (S) protein are located in its receptor-binding domain which is in direct contact with host cells; 4) MERS-CoV frequently transmitted back and forth between human and camel after it had acquired the human-camel infection capability. Together, these results suggest that potential recombination events might have happened frequently during MERS-CoV’s evolutionary history and the positive selection sites in MERS-CoV’s S protein might enable it to infect human.

Middle East Respiratory Syndrome coronavirus (MERS-CoV) is a novel beta-coronavirus with high pathogenicity, which imposes a serious threat to human health^1^. Substantial evidence has showed that MERS-CoVs have existed in central and east Africa for decades^2^,^3^, and have many natural hosts including two species of bats *(Neoromicia capensis* and *Vespertilio superans*), dromedary camel (*Camelus dromedarius*), and European hedgehog (*Erinaceus europaeus*)^4^,^5^,^6^,^7^. Yet it seemed that human MERS-CoV first isolated in Saudi Arabian in 2012, and then spread among humans worldwide^8^. Studies of prototype human MERS-CoV also suggested that the tMRCA (the time of most recent common ancestor) of human MERS-CoV can be traced back to about 2011^9^. Because the genome sequence of bat MERS-CoV is highly identical to that of human MERS-CoV, it has been indicated that human MERS-CoV might have a bat origin^10^, while there is also some evidence of camel-to-human MERS-CoV transmission^11^,^12^. Together, these studies proposed that zoonotic event may play a nontrivial part in MERS-CoV evolution and transmission.

Previous studies showed that recombination was common among the members of beta-coronavirus^13^,^14^. By joining the previously unlinked DNA, recombination event can create new viral strains which may be capable of infecting new hosts and evading host’s immuno-responses. The phylogenetic analysis showed that there were two main MERS-CoV clades—clade A and B, and clade B can be further divided into five phylogenetic groups^15^. There is evidence that recombination event had happened between group III and group V^15^. However, whether there was potential recombination event happened between the other groups or among multiple phylogenetic groups remains unclear.

The genome of MERS-CoV is over 30,000 nucleotides (nt) in length, which contains seven predicted open reading frames (ORFs) and four structural genes—spike (S), envelope (E), membrane (M), and nucleocapsid (N)^16^ (supplementary Fig. 1c). The surface located spike (S) protein of beta-coronaviruses is one of the major determinants in their cross-species transmission because it mediates the virus-receptor recognition and thereby activates viral infection process^17^. Additionally, the receptor binding domain (RBD) on S protein’s N-terminal is the key element for beta-coronavirus entering into host cells and the mutations in coronavirus’s RBD affects its infection and cross-species capability^18^,^19^. For example, the study of the spike protein from severe acute respiratory syndrome coronavirus (SARS-CoV), another beta-coronavirus with high pathogenicity, revealed four amino acid substitutions in its S protein receptor binding domain along with its switching host from civet to human^20^. Moreover, two amino acid substitutions in the S protein’s C-terminal of HKU4, a bat beta-coronavirus, enable its entry to human cells and the same amino acid substitutions are also found in MARS-CoV^21^. Furthermore, heptad repeat regions in C-terminal of MERS-CoV and related coronaviruses also play important roles in viral adaptive evolution^22^. In summary, those studies introduced above suggested that S protein plays a vital role in MERS-CoV’s cross-species transmissibility. However, the evolutionary mechanism of how MERS-CoV’s S and other proteins facilitated the cross-species transmission of MERS-CoV remains to be investigated.

Here, we performed a series of phylogenetic and bioinformatic analyses for MERS-CoVs. We systematically investigated the recombination events in MERS-CoVs, the potential transmission route of MERS-CoVs in five different host species and the evolutionary pressure of each MERS-CoV’s protein during cross-species transmission. Our study might offer some insight in explaining the possible mechanism in MERS-CoV’s adaptive evolution.

## Results

### Epidemic description and phylogenetic analysis of MERS-CoV

By far, the largest MERS-CoV outbreak is in Saudi Arabia and almost all human cases have a direct or indirect link to Arabian Peninsula. In this study, we collected 74 human MERS-CoV whole genome sequences from 9 countries (supplementary Table 1). The geographic distribution of these samples is shown in supplementary Fig. 1a. The majority of them are from the countries in Arabian Peninsula (78.4%, 58/74) and more than half of them are from Saudi Arabia (64.9%, 48/74) (supplementary Fig. 1a). The peak season for MERS is between April (26.5%) and may (25.0%) (supplementary Fig. 1b).

**Figure 1:**
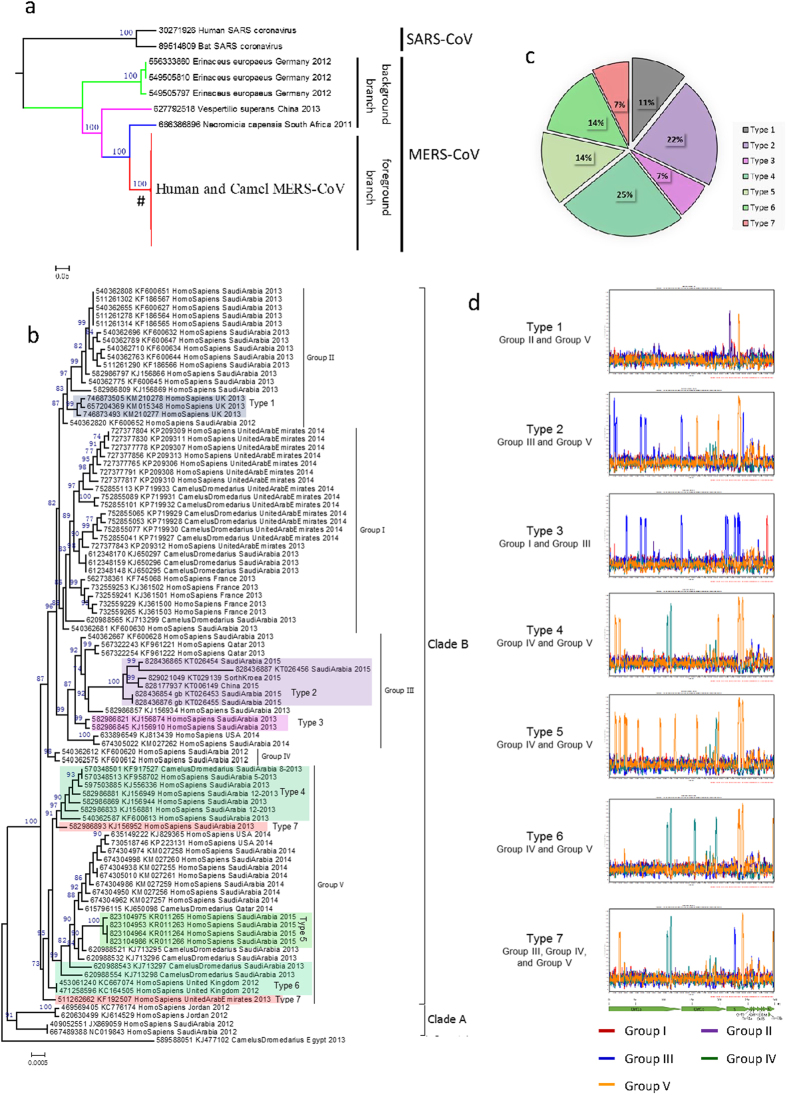
(**a**) Phylogenetic analysis of MERS-CoVs. The tree was constructed with maximum-likelihood method. SARS-CoVs are colored in black and serve as the outgroup to root the tree. Green, purple, blue and red represent hedgehog MERS-CoV group, bat *Vespertilio superans* MERS-CoV group, bat *Neoromicia capensis* MERS-CoV group, human-camel MERS-CoV group, respectively. (**b**) Phylogenetic analysis of human-camel MERS-CoVs. The tree was constructed with maximum-likelihood method. The camel MERS-CoV isolated in Egypt (GI: 589588051) is basal to clade A and B and serves as the outgroup to root the tree. Different shaded colors mean different potential recombinant types. (**c**) The percentage of potential recombinant type in all recombination strains. (**d**) The potential recombinant types. Red, purple, blue, green and yellow stand for consensus sequences of phylogenetic group I to V, respectively.

Based on the whole-genome alignment of our collected sequences (supplementary Table 1), we performed the phylogenetic analysis for these sequences with two SRAS-CoVs serving as the outgroup. Our phylogenetic tree shows that all camel and human MERS-CoVs are clustered together. The bat and hedgehog MERS-CoVs formed a basal paraphyletic group to all camel and human MERS-CoV clade (Fig. 1a). A single camel MERS-CoV isolated in Egypt (GI: 589588051) forms a single basal clade to human and the other camel MERS-CoVs (Fig. 1b). The human-camel MERS-CoV cluster can be further divided into two clades—clade A and clade B, as previously reported^16^. Clade A contains four human strains isolated in Jordan and Saudi Arabia while 70 human and 17 camel MERS-CoVs are mixed in clade B. There are five groups in clade B and we named them as group I to group V as the previous study^15^. There are 25, 17, 14, 2 and 29 MERS-CoV sequences in group I to group V, respectively (Fig. 1b).

### Recombination of MERS-CoV

We performed the recombination analysis on the collected full-length MERS-CoV sequences. We find that there are 28 of them experienced potential recombination events (30.4%, 28/92), including three camel MERS-CoVs and 25 human MERS-CoVs (supplementary Table 1). We divided 28 potential recombinant sequences into seven different types and named them as type 1 to type 7 (Fig. 1b–d, supplementary Table 1). Type 1 means the recombination happened between group II and group V, which includes 3 sequences and is about 11% of total recombinant sequences. Type 2 means the recombination happened between group III and group V, which includes 6 sequences (22%). Interestingly, the MERS-CoVs newly found in 2015 in South Korea and China are type 2 recombinants^15^,^23^. Type 3 means the recombination happened between group I and group III, which includes 2 sequences (7%). Type 4, 5 and 6 are the recombination happened between different genomic regions of group IV and group V, which include 7, 4 and 4 sequences (25%, 14% and 14%), respectively. Type 7 is the recombination happened among three groups (group I, IV and V), which includes 2 sequences (7%). Our phylogenetic analysis showed type 1 belongs to phylogenetic group II while type 2 and 3 belong to phylogenetic group III, and type 4 to 7 belong to phylogenetic group V. There is no recombination found in phylogenetic group I and group IV (Fig. 1b). We also reconstructed the phylogenetic tree using non-recombinant sequences only and found that its topology is consistent with the tree based on all sequences (supplementary Fig. 2). We also performed the SNP (single-nucleotide polymorphisms) analyses for each recombinant types and found the large recombination segments in type 2, 4, 6, 7 are conspicuous but in type 1, 3, 5 are obscure (supplementary Fig. 3).

### Adaptive selection analysis for MERS-CoV proteins

**Figure 2:**
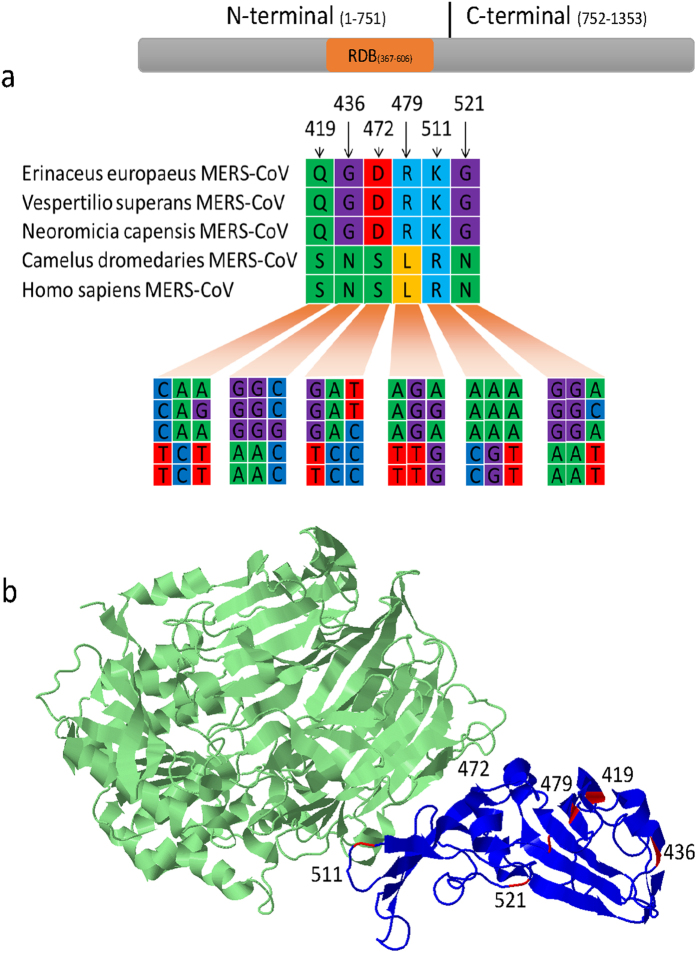
(**a**) Detected positive selection sites in S protein’s receptor binding domain and their corresponding codons. (**b**) Human receptor dipeptidyl peptidase 4 is colored in green and S protein’s receptor binding domain is colored in blue. The detected positive selection sites are marked with red color and black numbers.

In order to explore the selection pressure on the MERS-CoV proteins when it transmitted from animal host to human, we performed the adaptive evolution analyses for all MERS-CoV protein in absence of recombinant strains. Firstly, we set camel and human MERS-CoVs as the foreground branch and bat and hedgehog MERS-CoVs as the background branch to preform branch-site test in CODEML of PAML program (see Fig. 1a). The strong positive selection is detected in spike (S) glycoprotein between these two branches (p < 0.001), while there is no significant positive selection in the other MERS-CoV genes (Table 1). We find nine positive selection sites in MERS-CoV spike (S) glycoprotein and eight of them are statistically significant (Table 1). Six significant positive selection sites are located in the receptor binding domain of S protein (Fig. 2a). We utilized a published crystal structure (PDB ID 4L72 in RCSB Protein Data Bank), the receptor binding domain (RBD, aa 367-606, Fig. 2b) of MERS-CoV spike glycoprotein complexed with the human receptor dipeptidyl peptidase 4 (DDP4), to demonstrate their locations in a 3D environment (Fig. 2b). The receptor binding domain of MERS-CoV S protein can be further divided into a receptor-binding sub-domain and a core sub-domain. Two significant positive selection sites, K511R and G521N, are in the receptor-binding sub-domain and K511R is in direct contact with human receptor DDP4. Q419S, G436N, D472S and R479L are in the core sub-domain. Moreover, we also detected a positive selection site in S protein’s c-terminal, L775S. Secondly, we screened the positive selection sites among human-camel MERS-CoVs (Table 2). Five significant positive selection sites are found in ORF 8b, M protein, N protein, and S protein (Table 2). Two of them are located in N proteins and one of them are located in M, S or open reading frame 8b (ORF8b), respectively.

### Substitution rate analysis for MERS-CoV proteins

The MERS-CoV genome contains 11 open reading frames (supplementary Fig. 1c). The nucleotide substitution rate of each open reading frame and the whole-genome sequence was estimated for camel and human MERS-CoVs (Table 3). The genome-wide average nucleotide substitution rate of camel and human MERS-CoVs was 4.81 × 10^−4^ substitutions per site per year. Open reading frame 3 (ORF3) has the fastest substitution rate while ORF5 has the slowest substitution rate. The ORF4b, nucleocapsid (N) glycoprotein, and spike (S) glycoprotein, have a similar substitution rate which is faster than the whole-genome substitution rate.

### Transmission analysis for MERS-CoVs

**Figure 3:**
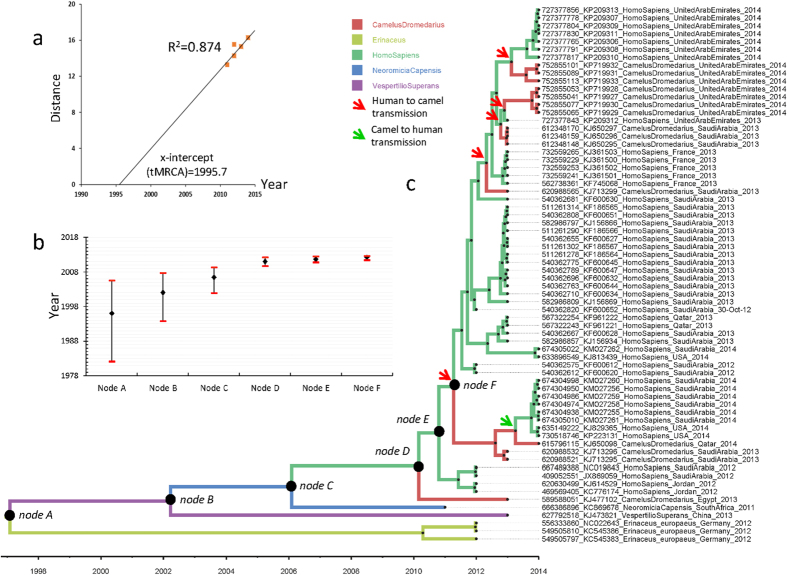
(**a**) Root to tip analysis shows the x-intercept (tMRCA) is ~1995 with an R^2^ of 0.874. (**b**) Original time and 95% HPD for node A to F. (**c**) Transmission analysis based on MCC method. Blue, red, yellow, green and cyan represent camel MERS-CoV, human MERS-CoV, hedgehog MERS-CoV, bat *Neoromicia capensis* and MERS-CoV and bat *Vespertilio superans* MERS-CoV. Node A to F are marked with black filled circle. Green arrows point out camel-to-human transmission and red arrows point out human-to-camel transmission in clade B.

In order to study the temporal and spatial pattern of MERS-CoV transmission, a maximum clade credibility (MCC) tree was constructed using MERS-CoV whole genome sequences without recombinant strains (Fig. 3c). The ancestral host state with time reference was estimated for each tree node and marked with different colors. We named six important nodes in MERS-CoV divergence on MCC tree for node A to F (Fig. 3c). The possible transmission time for each node and its 95% highest posterior density (HPD) are shown in Fig. 3b. We found that the origin time of human-camel MERS-CoV is relatively late (node D). Furthermore, the tMRCA for clade B is in ~2012 (Fig. 3b, node F) and clade A and clade B are divergent in ~2011 (Fig. 3b, node E). Interestingly, the MCC tree shows that there are six cross-species transmission events with high posterior probabilities in clade B. Five of them are human-to-camel transmission events and one of them are camel-to-human transmission events (Fig. 3c). Additionally, with the MERS-CoV of human/camel and bat/hedgehog MERS-CoV together, we inferred the ancient MERS-CoV exists for decades of years (Fig. 3b,c). The tMRCA of the MERS-CoVs for *Vespertilio superans, Neoromicia capensis* or *Erinaceus europaeus* can be traced back to 2006 (node C), 2003 (node B) and 1996 (node A), respectively. Before the emergence of human-camel MERS-CoV, the estimated tMRCA for all MERS-CoVs appeared in ~1996 (Fig. 2c, node A). We also preformed root-to-tip analysis using the consistent dataset (Fig. 3a), and the result shows that the origin time of tMRCA is in ~1995 with high statistical supports (R^2^ = 0.874, p value < 0.001). Together these results suggest that the ancient MERS-CoV should have existed for decades in animal host and got the ability to infect human or camel recently.

## Discussion

MERS-CoV belongs to coronavirus, beta-coronavirus, lineage C. Since it was discovered in 2012, MERS-CoV has attracted extensive attention due to its human-to-human infection capability and high mortality rate. Recombination events have been confirmed in human MERS-CoV^23^. The fact that MERS-CoV can be found in multiple species proposes its cross-species transmissibility^4^,^5^,^6^,^7^,^11^. By far, the evolutionary details of how MERS-CoV transmitted to human are still unknown. Based on the most comprehensive collection of MERS-CoV genome sequences so far, we tried to elucidate the evolution and transmission of MERS-CoV among different species.

MERS-CoV has been reported in five species including European hedgehog, two species of bats, dromedary camel, and human. We used the ML method to reconstruct the whole-genome phylogenetic tree of MERS-CoVs isolated from these species. The ML tree shows that the hedgehog MERS-CoVs are basal to all the other MERS-CoVs and two bat MERS-CoVs are basal to camel and human MERS-CoVs. This result suggests that the ancestor of camel and human MERS-CoVs may be from other animal host, such as the hedgehog or bat. We also reconstructed the phylogenetic tree of MERS-CoV using NJ method or based on each MERS-CoV protein. These trees show a consistent topology, which proposes that the phylogenetic relationship estimated in our study is credible (supplementary Fig. 5).

We divided clade B into five groups as pervious study to detect the recombination of MERS-CoV^15^. Because the evolutionary distances among MERS-CoVs are close (Table 3), no large segment recombination could be detected among them. Thus, according to discontinuous recombination segments, we defined potential recombination events in MERS-CoVs. This method has been used in the previous study to label potential recombination event^24^. In our study, we found 28 strains form seven recombinant types, which took more than 30% of all isolated MERS-CoVs in human and camel. Among them, we found 26 strains in six recombinant types (type 1 to type 6) between two phylogenetic groups and two strains in one type (type 7) among three phylogenetic groups. For now, the recombination of MERS-CoV was confirmed in previous study, but no report about the recombination among more than two groups of MERS-CoV. Interestingly, most recombinant types (type 1, 2, 4, 5, 6 and 7) are related to group V and they make up 92.9% of total recombinant strains (26/28). The result suggests that recombination events might happen frequently and the recombinant types involving group V might happen broadly. Additionally, multiple recombination events indicate that double infection and super infection likely existed during the transmission history of MERS-CoV. We failed to detect possible large recombination segments in type 1, 3 and 5. By comparing the SNPs (single-nucleotide polymorphisms) of reference sequences with recombinant sequences, we reckoned that specific nucleotide mutation might influence the results of recombination analysis. This problem can be solved by discovering more MERS-CoV sequences or developing more detailed genotype classification for MERS-CoVs in the future. We also performed phylogenetic analyses for potential recombinant region and got similar results (supplementary Fig. 3).

Interestingly, the East Asian MERS-CoV strains (China and South Korea) belong to type 2 recombinant and the previous study show that their tMRCA might be a result of potential recombination event^23^, which indicates the recombinant strains have transmitted broadly. Moreover, one recombinant MERS-CoV lineage has led to the large-scale outbreak in both camel and human^26^. It proposes that recombinant MERS-CoVs have experienced cross-species infection. Additionally, our study reveals that the number of recombinant strains is large and the potential recombinant types are abundant. Together these findings highlight that we should take more attention to recombinant MERS-CoV transmission.

Although how the MERS-CoV transmitted from its natural host to human is still unknown, it is confirmed the MERS-CoV have been found in many animal hosts, such as bats and hedgehog. To study the evolutionary pressure on each MERS-CoV’s protein during its potential cross-species transmission, we conducted a comprehensive scan for positive selection sites in MERS-CoV’s proteins. Recombinant strains were excluded in this analysis. We set camel-human MERS-CoVs as the foreground branch and hedgehog-bat MERS-CoV as the background branch and estimated the relative evolutionary pressure on the foreground branch compared to the background branch. We only found that MERS-CoV’s S protein underwent strong positive selection and there are nine significant positive selection sites in S protein. It suggests that S protein was under strong evolutionary pressure during the transmission from its natural host to human. Among significant positive selection sites, six of them are located in S protein’s receptor binding domain (RBD). RBD is crucial for virus to enter host cells and it comprises of one binding region and one core region. Based on the RBD’s 3D model, we find that two sites are located in the binding region of RBD, which suggests that these amino acid substitutions might change MERS-CoV’s binding capability to host cells and thus facilitate its cross-species transmission. The other four sites are located in the core region of RBD. These amino acid substitutions might change the structure of core region and indirectly influence MERS-CoV’s cross-species capability. In order to eliminate the sample bias between human/camel group and bat/hedgehog group, we also did random sampling for the 68 non-recombinant human and camel sequences. We tried 10, 20 and 50 random sampling, respectively. Using random sampled sequences together with bat and hedgehog MERS-CoV sequences, we performed branch-site analyses and got the same results as our aforementioned (data not show).

We also estimated the nucleotide substitution rates of camel-human MERS-CoVs to investigate its evolutionary dynamics after it infected camel and human. Our estimated nucleotide substitution rate for MERS-CoV’s whole genome is 4.81 × 10^−4^, which is slower than the previous estimation^9^. One explanation for the phenomenon is that we used a larger dataset than the previous study, which includes all available MERS-CoV whole genome sequences. Our estimated confidence interval of the substitution rate of MERS-CoV genome is largely overlapped with the result from another study^16^. The estimated nucleotide substitution rates show that four proteins experienced the accelerated evolution.

Through evolutionary pressure analysis, we found camel-human MERS-CoV’s four proteins underwent positive selection and detected five significant positive selection sites. One of them is located in M proteins. There is evidence that M proteins are powerful interferon antagonist^26^, which proposes that the evolutionary pressure on M proteins are from host’s immune system. Two out of five significant sites are found in N protein which is fundamental for MERS-CoV self-assembly. Coronavirus N protein is able to bind to different host cell proteins and demonstrated to have various functions, one of which is also counteracting host interferon as shown in SARS-CoV^27^,^28^,^29^,^30^. It is reasonable to speculate that MERS-CoV N protein under intensive selection because its functions were similar to those of SARS-CoV N protein. The results above suggest that the arm race between MERS-CoV proteins and host’s immune system might be the main evolutionary driving force behind MERS-CoV’s adaptive evolution after it began to infect camel and human. MERS-CoV spike (S) glycoprotein evolves slightly faster than the genome-wide average rate, which indicates that the nucleotide substitution rate of MERS-CoV S protein still maintains a fast speed even after it crossed the species boundary. The positive selection site we found in MERS-CoV S protein with site-specific test is identical to the previous study’s result^3^. This site is located in heptad repeats 1 which is a key component in membrane fusion architecture and required for MERS-CoV entering host cells^31^.

In absence of recombinant strains, we performed the MCC analysis using MERS-CoV whole-genome sequences in order to infer the time and source species when MERS-CoV crossed the species boundary. The topology of the MCC tree is highly congruent with that of the whole-genome phylogenetic tree. We defined six nodes (A–F) to explain transmission. The posterior probability for the ancestral sate of node A, B or C is not very high in our MCC analysis and the 95% highest posterior density (HPD) of the divergence time for these three nodes is quite long. So these results are weak for demonstrating the exact origin time or ancestor state of MERS-CoV. However, these estimations still provided the evidence that the ancestor MERS-CoV should have been infected a number of animal hosts, such as bat or hedgehog, for decades of years (supplementary Fig. 4). The x-intercept (tMRCA) in root-to-tip is ~1995 with high statistical supports, which is close to the estimated time of tMRCA in MCC analysis. This hypothesis is in agreement with the result of serological studies^2^,^3^. The appearance of the common ancestor of human-camel MERS-CoV is in 2010 and the appearance of the tMRCA of clade A and B is in 2011, which are exactly the same as the previous report^16^. In clade B, we detected five possible human-to-camel transmission events and one camel-to-human transmission event. It suggests that MERS-CoV frequently transmitted back and forth between human and camel after it acquired the capability of infecting both hosts. Actually, there is at least one confirmed case of camel-to-human MERS-CoV transmission^32^.

## Conclusion

In conclusion, we found that potential recombination events are common in MERS-CoV’s evolutionary history and potential recombinant MERS-CoVs can be divided into seven types. The amino acid sites under positive selection in MERS-CoV S protein, especially those in its receptor binding domain, might have facilitated its cross-species transmission from animal host to human. We detected the strong positive selection in four proteins of camel-human MERS-CoVs, which indicates that they probably experienced strong adaptive evolutionary pressure from host’s immune system. Additionally, we also found six possible cross-species transmission cases between human and camel. Our study investigated the evolutionary dynamics of MERS-CoV, which shall provide a basis for MERS-CoV control and treatment.

## Materials and Methods

### Sequence data

The complete genomic nucleotide sequences of 91 MERS-CoVs and two SARS-CoVs were downloaded from NCBI nucleotide database. Among 91 MERS-CoV genomic sequences, 68 of them are from human, 18 of them are from dromedary came, two of them from two bat species *Neoromicia capensis* and *Vespertilio superans*, and 3 of them are from European hedgehog *Erinaceus europaeus*. Two SARS-CoV genomic sequences are from human and bat *Rhinolophus ferrumequinum*, respectively. We used sequences 453061240 as reference to extract open reading frames from each MERS-CoV genome in this study.

### Genomic sequence alignment and phylogenetic analysis

Total 93 collected genomic sequences were aligned using the MUSCLE software with default parameters^33^. ClustalW and MAFFT used to validate the MUSCLE result^34^,^35^. Alignments were refined manually in Bioedit (http://www.mbio.ncsu.edu/BioEdit/BioEdit.html). Only unambiguously aligned positions were used for subsequent phylogenetic analyses (supplementary files). We used the JmodelTest 3.1 to estimate the best nucleotide substitution model for our alignment^36^, which is GTR+I+G. We used the PHYML 3.1 to perform the phylogenetic analysis for 93 collected genomic sequences based on their genomic sequence alignment^37^. The branch support values were calculated with Shimodaira-Hasegawa test integrated in PHYML.

### Recombination analysis

In clade B, we estimated the consensus sequences for every phylogenetic group using cons tool in EMBOSS explorer (http://bioinfo.nhri.org.tw/cgi-bin/emboss/cons). Five consensus sequences were set as reference and every sequence in clade B was used as the query to detect the possible recombination using Simplot software^25^. The window is set to 200 bp and the step is set to 20 bp.

### Positive selection analysis

We extracted the coding region of each MERS-CoV protein using MERS-CoV 453061240 strain as a reference template. The CODEML program implemented in PAML 4.7 package was used to detect the positive selection in the codon alignment of each MERS-CoV protein set^38^. In the branch-site model, the group of human-camel MERS-CoVs was set to be foreground, the group of bat-hedgehog MERS-CoVs was set to be background, and model A with estimated ω value was compared with the null model (model A’) with fixed ω value. To reduce the bias from sample size, we performed random sampling on 68 human and camel MERS-CoVs (clade A and B) which are all non-recombinant sequences. We used 10, 20 and 50 as the random sample size with and without replacement. The random sampled sequences together with bat and hedgehog MERS-CoVs were used to generate the datasets for branch-site model analysis as aforementioned method (script see supplementary files). Moreover, for each random sample size, we repeatedly drew five times in order to make results robust.

We also used the site-specific model to detect positive selection in the human-camel clade. The site-specific model was performed by comparing the models M2a (positive selection) and M8 (beta & ω) vs. the null models M1a (nearly neutral) and M7 (beta), respectively. Evolutionary distance of each protein was estimated using MEGA 6^39^.

### 3D structure display

The crystal structure of the receptor binding domain (RBD) of MERS-CoV spike (S) glycoprotein in complex with the human receptor dipeptidyl peptidase 4 (DDP4) was displayed using Jmol (Jmol: an open-source Java viewer for chemical structures in 3D. http://www.jmol.org/).

### Transmission analysis

We reconstructed the maximum clade credibility (MCC) tree using a MCMC (Markov Chain Monte Carlo) method implemented in the BEAST v1.8.2 package^40^. We estimated the transmission of MERS-CoV among different hosts or geographic areas. The sampling time and host/geographic location of each sequence were also used in analysis. The nucleotide substitution rates and the origin time of most recent common ancestor (MRCA) on various nodes of MCC tree were also estimated using the BEAST package. A relaxed molecular clock with an uncorrelated log-normal distribution, and a constant population size model were used in Bayesian coalescence analysis. According to the outcome of JmodelTest3.1, the GTR+Gamma+I model of nucleotide substitution was employed in MCC analysis. Statistical uncertainty in parameter estimations was reflected by the 95% highest posterior density (HPD) values. MCMC analysis was run for 500/100 million generations for hosts/geographic transmission with sampling every 50,000/10,000 generations to achieve parameter convergence and adequate effective sample sizes (ESS > 200). We summarized the trees using TreeAnnotator implemented in the BEAST v1.8.2 package. The initial 25% samples were discarded as burn-in, leaving 75% trees per run to produce the consensus tree. Root-to-tip analysis was performed with Path-O-Gen software (version 1.4, http://tree.bio.ed.ac.uk/software/pathogen/).

## Additional Information

**How to cite this article**: Zhang, Z. *et al*. Evolutionary Dynamics of MERS-CoV: Potential Recombination, Positive Selection and Transmission. *Sci. Rep*. **6**, 25049; doi: 10.1038/srep25049 (2016).

## Supplementary Material

Supplementary Information

## Figures and Tables

**Table 1:** Branch-site tests for positive selection on spike (S) protein by comparing foreground MERS-CoV (from human and camel) with background MERS-CoV (from bat and hedgehog).

	lnL MA’	lnL MA	P value	Positively selected sites (PSS)#	
S	−16251.74	−16207.52	<0.001	141R, 270Y*, 419S**, 436N*, 472S*, 479L*, 511R**, 521N*, 775S*	
N	−4449.82	−4449.64	p > 0.05	NULL	
M	−2051.31	−2051.14	p > 0.05	NULL	
E	−703.78	−703.17	p > 0.05	8Q, 11S, 56I	
8	−1022.67	−1018.75	p < 0.01	NS	
5	−2583.27	−2583.27	p > 0.05	NULL	
4b	−2659.78	−2659.76	p > 0.05	NULL	
4a	−1056.27	−1056.27	p > 0.05	NULL	
3	−1165.51	−1165.50	p > 0.05	NULL	

^#^Only PSSs with post posterior probability (PP) > 0.9 are shown. *Means PP > 0.95, **means PP > 0.99. P value was estimated using χ^2^ tests with with degree of freedom = 1.

**Table 2:** Site-specific tests for positive selection on different MERS-CoV proteins in camel and human.

Protein (orf)	ln LM1	ln LM2	P value	PSS (BEB)#	ln LM7	ln LM8	P value	PSS (BEB)#
1a	−20604.16	−20602.67	P > 0.05	NS	−20604.16	−20602.67	P > 0.05	NS
1b	−10846.50	−10846.48	P > 0.05	NS	−10846.50	−10846.48	P > 0.05	NS
3	−561.10	−560.43	P > 0.05	86P	−561.11	−560.43	P > 0.05	86P
4a	−521.41	−520.06	P > 0.05	NS	−521.41	−520.06	P > 0.05	NS
4b	−1018.79	−1018.53	P > 0.05	NS	−1018.79	−1018.53	P > 0.05	NS
5	−1062.76	−1062.75	P > 0.05	NS	−1062.76	−1062.75	P > 0.05	NS
8b	−1071.00	−1053.13	P<0.05	79Q**	−1071.00	−1053.13	P < 0.05	79Q**
E	−321.39	−321.39	P > 0.05	NULL	−321.39	−321.39	P > 0.05	NULL
M	−965.70	−958.93	P < 0.05	69V**	−965.70	−958.93	P < 0.05	69V**
N	−1903.63	−1896.13	P < 0.05	144S, 366K	−1903.62	−1896.14	P < 0.05	126D, 144S*, 366K*
S	−6299.02	−6293.81	P < 0.05	1020Q*	−6299.06	−6293.82	P < 0.05	1020Q*

^#^Only PSSs with post posterior probability (PP) > 0.9 are shown. *Means PP > 0.95, **means PP > 0.99. P value was estimated using χ^2^ tests with with degree of freedom = 2.

**Table 3:** Evolutionary distance, nucleotide substitution rate and coefficient of variation of substitution rate for human and camel MERS-CoV proteins.

	Distance (10^−2^)	Substitution rates (10^−4^)	Coefficient of Variation
1a	0.2 ± 0.005	4.06	0.45
1b	0.1 ± 0.001	3.93	0.33
3	0.7 ± 0.2	50.30	3.39
4a	0.5 ± 0.2	3.87	0.86
4b	0.2 ± 0.1	7.95	3.85
5	0.4 ± 0.1	1.42	1.48
8b	0.4 ± 0.1	3.54	2.70
E	<0.001	3.20	0.5
M	0.1 ± 0.003	1.75	2.00
N	0.2 ± 0.08	9.77	2.42
S	0.3 ± 0.01	7.64	0.22
Whole genome	0.2 ± 0.01	4.81	0.43
