# Supplementary material for: Evolutionary Dynamics of MERS-CoV: Potential Recombination, Positive Selection and Transmission
Source: Sci Rep. 2016 May 4;6:25049. doi: 10.1038/srep25049 (PMC4855236; doi:10.1038/srep25049)
Supplement: Supplementary Information [file srep25049-s1.doc]

# Supplementary information

# Evolutionary Dynamics of MERS-CoV: Potential Recombination, Positive selection and Transmission.

Zhao Zhang^1, *^, Libing Shen^1, *^ and Xun Gu^1, 2, #^

Author affiliations:

^1^State Key Laboratory of Genetic Engineering and MOE Key Laboratory of Contemporary Anthropology, School of Life Sciences, Fudan University, Shanghai, 200433, PR China

^2^Department of Genetics, Development, and Cell Biology, Iowa State University, Ames, IA, 50011, USA

^*^These authors contributed equally to this work.

^#^ Corresponding author:

XunGu, State Key Laboratory of Genetic Engineering and MOE Key Laboratory of Contemporary Anthropology, School of Life Sciences, Fudan University, Shanghai, 200433, PR China. E-mail: xgu@iastate.edu Tel: 0086-21-5163 0613.


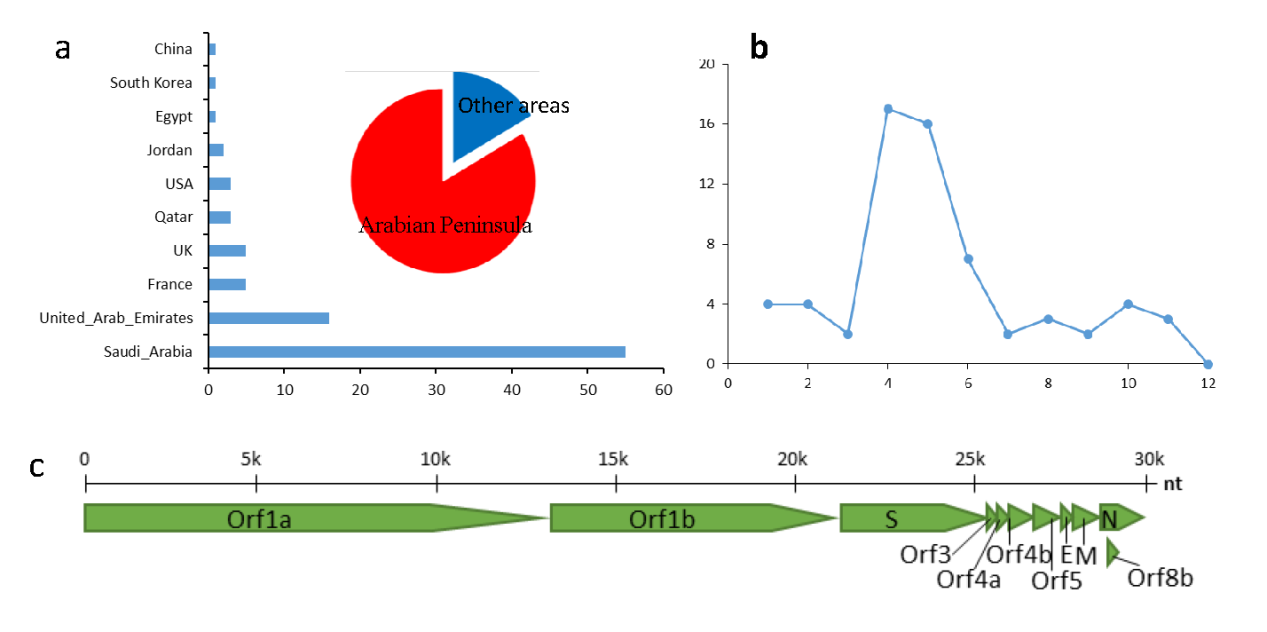


Supplementary figure 1 .

a. Distribution of MERS-CoV cases in different countries and regions.

b. Distribution of MERS-CoV cases in different months within a year.

c. Genome organization of MERS-CoV.


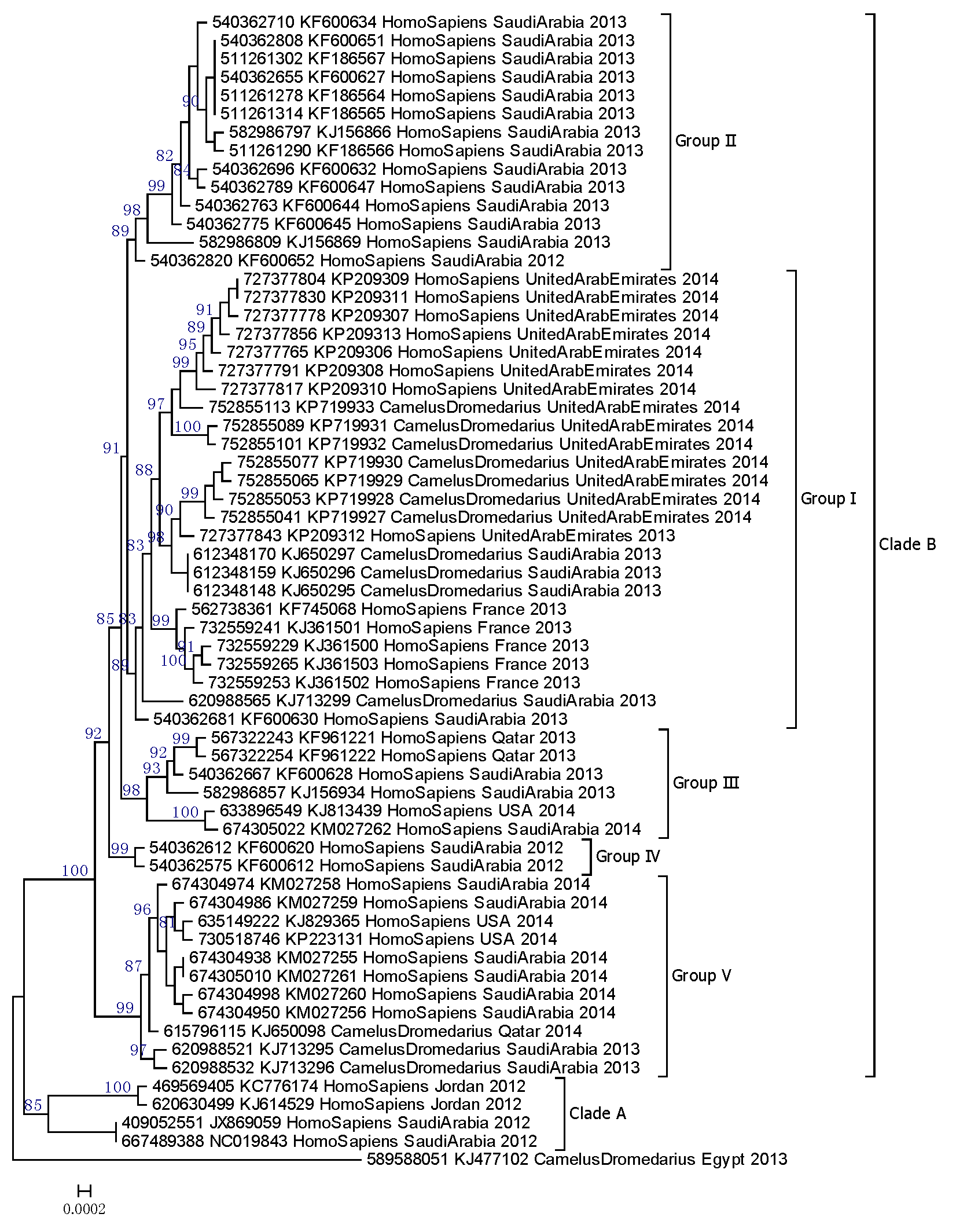


Supplementary figure 2

Phylogenetic tree of MERS-CoV in absence of potential recombinant strains.

Type 1


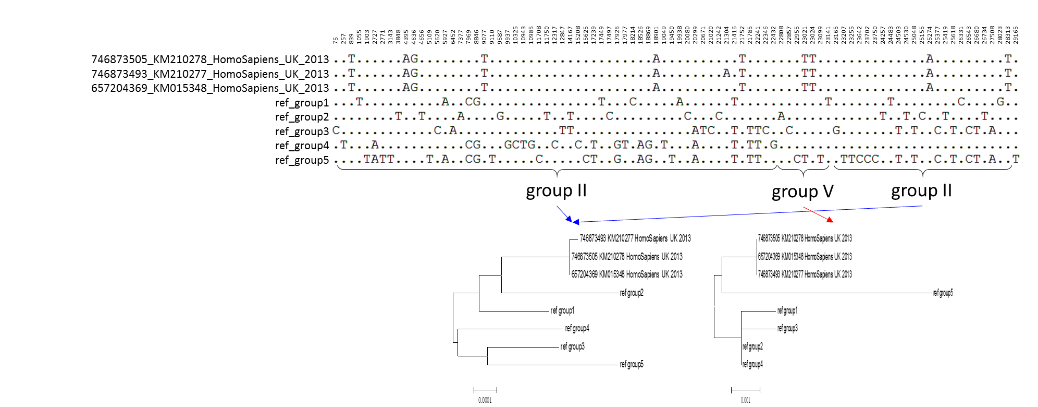


Type 2


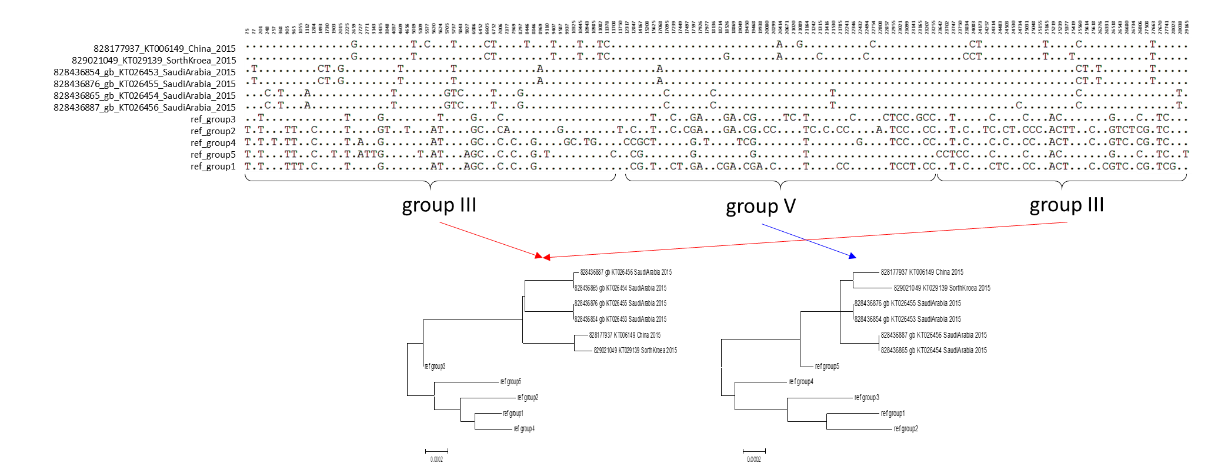


Type 3


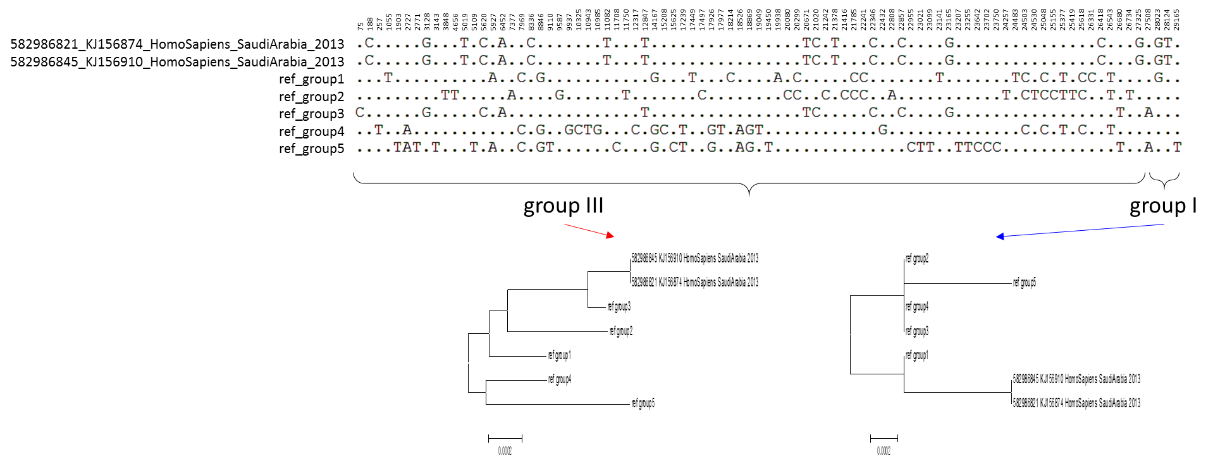


Type 4


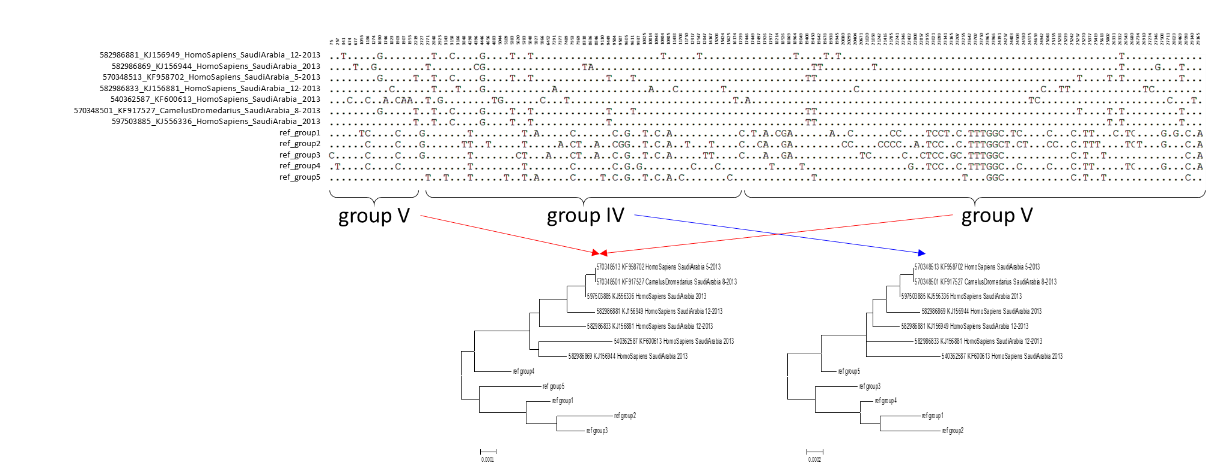


Type 5


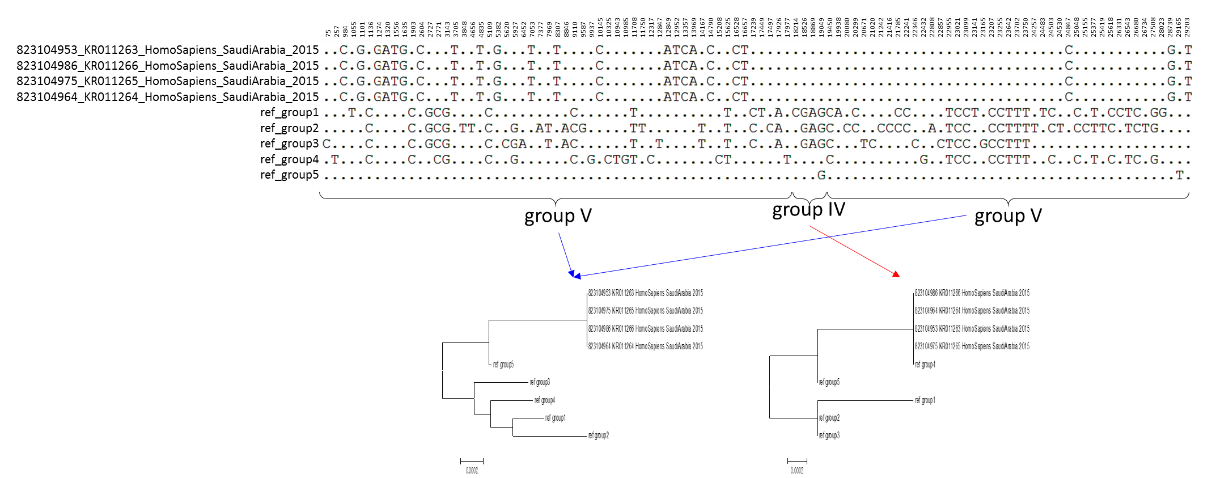


Type 6


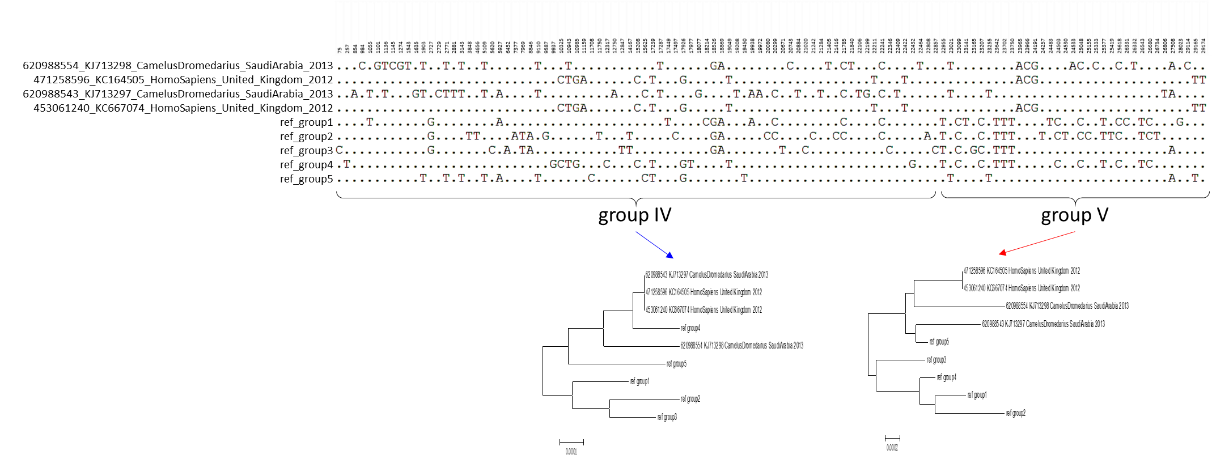


Type 7


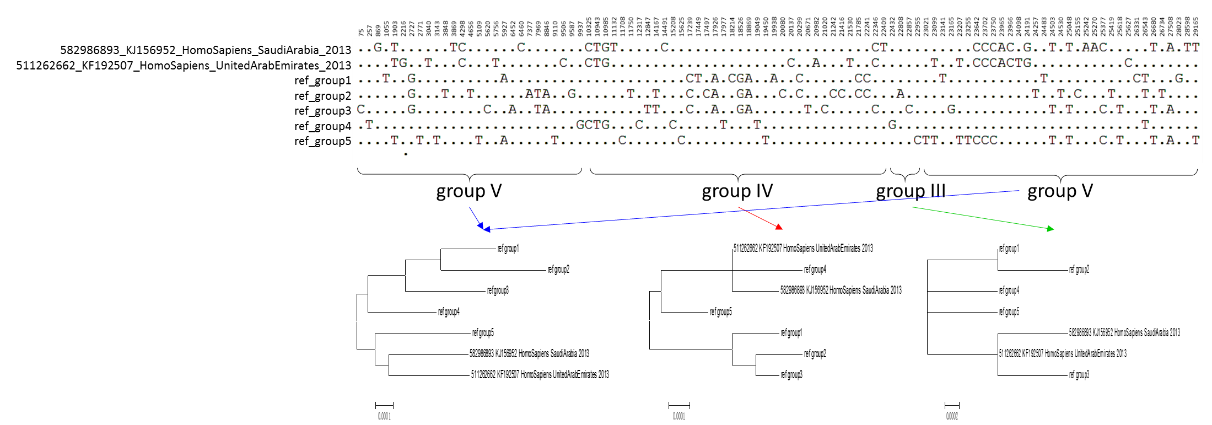


Supplementary figure 3

SNP analyses and phylogenetic analyses of possible recombination regions for the seven potential recombinant types.


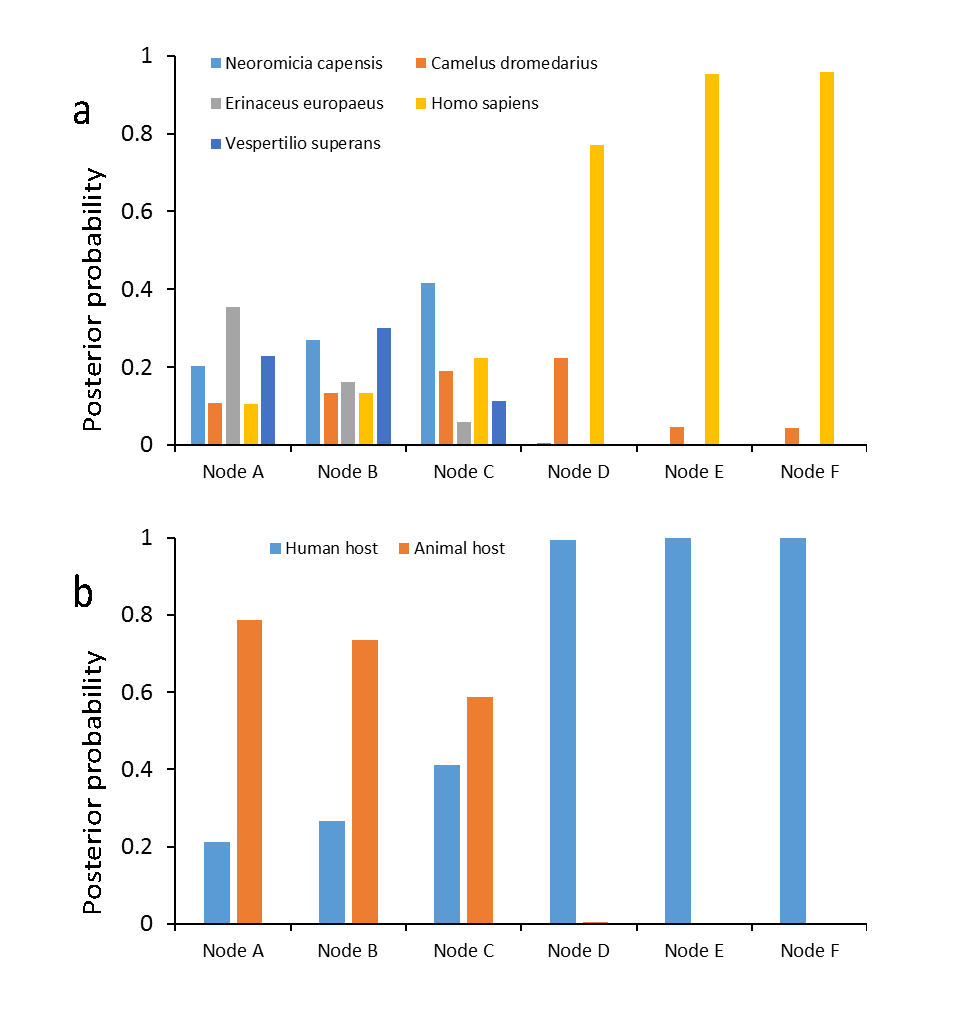


Supplementary figure 4.

Posterior probability of ancestor state for MERS-CoV ancestor. Human host means the ancestor state is human or camel, animal host means the ancestor state is bats or hedgehog.


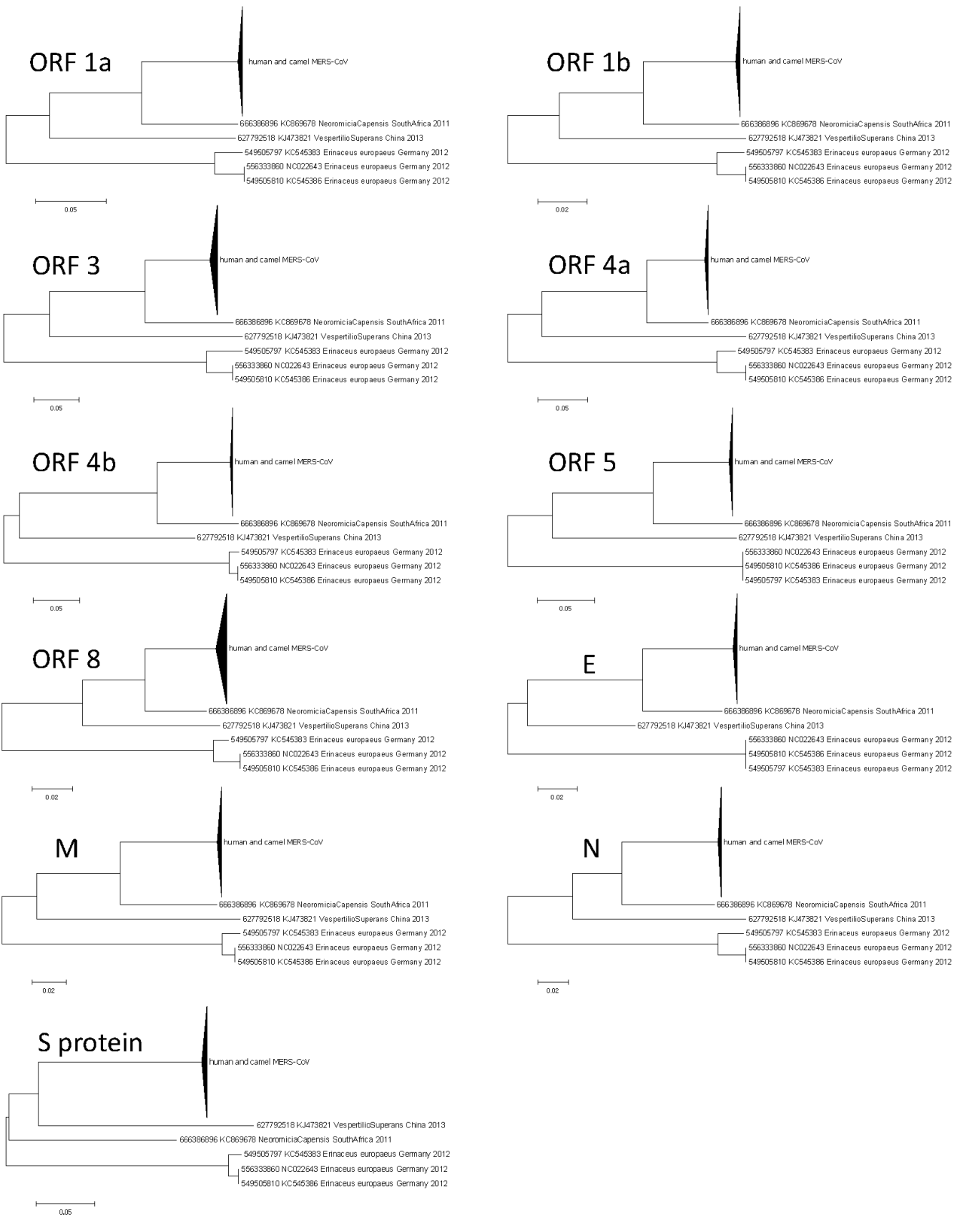


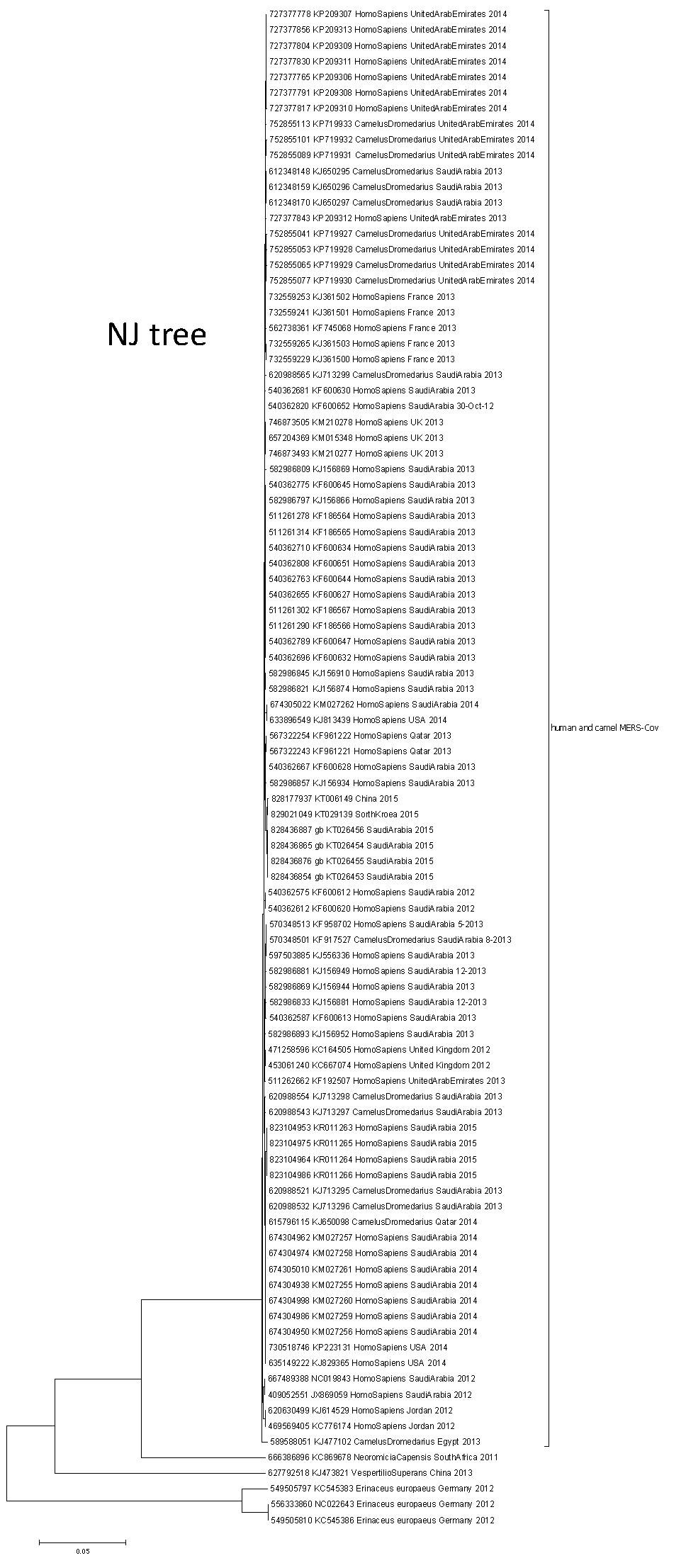


Supplementary figure 5

Phylogenetic tree of each MERS-CoV proteins constructed with ML method and Phylogenetic tree based on whole genome and constructed with NJ method. Their topology structures are consistent with figure 1a.

Supplementary table 1. Isolated time, geographic location, host, accession number, and recombination analyses results for every collected strains.

| *GI* | *ACCESSION* | phylogenetic group | *Host* | *Address* | Isolate time | recombination type | Simplot result |
| --- | --- | --- | --- | --- | --- | --- | --- |
| *666386896* | KC869678 |  | Neoromicia_capensis | South_Africa | 2011 |  |  |
| *627792518* | KJ473821 |  | Vespertilio_superans | China | 2013 |  |  |
| *549505797* | KC545383 |  | Erinaceus_europaeus | Germany | 2012 |  |  |
| *549505810* | KC545386 |  | Erinaceus_europaeus | Germany | 2012 |  |  |
| *556333860* | NC022643 |  | Erinaceus_europaeus | Germany | 2012 |  |  |
| 409052551 | JX869059 | clade A | Homo_sapiens | United_Kingdom | 19-Sep-12 |  |  |
| 469569405 | KC776174 | clade A | Homo_sapiens | United_Kingdom | 11-Sep-12 |  |  |
| 589588051 | kj477102 | clade A | Camelus_dromedarius | Saudi_Arabia | 6-Nov-13 |  |  |
| 620630499 | kj614529 | clade A | Homo_sapiens | Saudi_Arabia | 13-Nov |  |  |
| 667489388 | nc019843 | clade A | Homo_sapiens | Saudi_Arabia | 2014 |  |  |
| 453061240 | KC667074 | 5 | Homo_sapiens | Jordan | 12-Apr | type6 |  |
| 471258596 | KC164505 | 5 | Homo_sapiens | Saudi_Arabia | 1-May-13 | type6 | 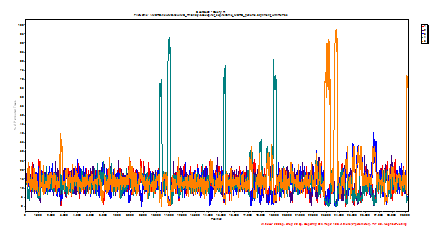 |
| 511262662 | kf192507 | 5 | Homo_sapiens | Saudi_Arabia | 23-Oct-12 | type7 |  |
| 540362587 | kf600613 | 5 | Homo_sapiens | Saudi_Arabia | 19-Jun-12 | type4 |  |
| 570348501 | kf917527 | 5 | Camelus_dromedarius | Saudi_Arabia | 5-Nov-13 | type4 |  |
| 570348513 | kf958702 | 5 | Homo_sapiens | Saudi_Arabia | 2-May-13 | type4 |  |
| 582986833 | kj156886 | 5 | Homo_sapiens | Saudi_Arabia | 15-Aug-13 | type4 |  |
| 582986869 | kj156944 | 5 | Homo_sapiens | Saudi_Arabia | 12-Jun-13 | type4 |  |
| 582986881 | kj156949 | 5 | Homo_sapiens | Saudi_Arabia | 1-Mar-13 | type4 |  |
| 582986893 | kj156952 | 5 | Homo_sapiens | Egypt | 2013 | type7 |  |
| 597503885 | kj556336 | 5 | Homo_sapiens | Saudi_Arabia | 30-Dec-13 | type4 |  |
| 615796115 | kj650098 | 5 | Camelus_dromedarius | Jordan | 2012 |  |  |
| 620988521 | kj713295 | 5 | Camelus_dromedarius | Saudi_Arabia | 13-Nov |  |  |
| 620988532 | kj713296 | 5 | Camelus_dromedarius | Saudi_Arabia | 13-Nov |  |  |
| 620988543 | kj713297 | 5 | Camelus_dromedarius | Saudi_Arabia | 13-Nov | type6 |  |
| 620988554 | kj713298 | 5 | Camelus_dromedarius | Saudi_Arabia | 13-Nov | type6 | 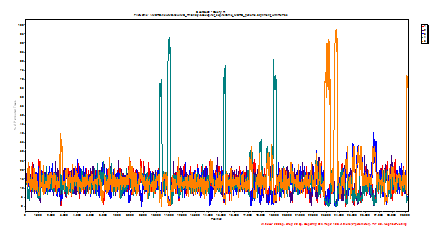 |
| 635149222 | kj829365 | 5 | Homo_sapiens | United_Kingdom | 10-Feb-13 |  |  |
| 674304938 | km027255 | 5 | Homo_sapiens | Saudi_Arabia | 2014 |  |  |
| 674304950 | km027256 | 5 | Homo_sapiens | Saudi_Arabia | 7-Apr-14 |  |  |
| 674304962 | km027257 | 5 | Homo_sapiens | Saudi_Arabia | 12-Apr-14 |  |  |
| 674304974 | km027258 | 5 | Homo_sapiens | Saudi_Arabia | 14-Apr-14 |  |  |
| 674304986 | km027259 | 5 | Homo_sapiens | Saudi_Arabia | 21-Apr-14 |  |  |
| 674304998 | km027260 | 5 | Homo_sapiens | Saudi_Arabia | 15-Apr-14 |  |  |
| 674305010 | km027261 | 5 | Homo_sapiens | Saudi_Arabia | 22-Apr-14 |  |  |
| 730518746 | kp223131 | 5 | Homo_sapiens | France | 26-Apr-13 |  |  |
| 823104953 | KR011263 | 5 | Homo_sapiens | Saudi_Arabia | 21-Jan-15 | type5 |  |
| 823104964 | KR011264 | 5 | Homo_sapiens | Saudi_Arabia | 26-Jan-15 | type5 |  |
| 823104975 | KR011265 | 5 | Homo_sapiens | Saudi_Arabia | 6-Jan-15 | type5 |  |
| 823104986 | KR011266 | 5 | Homo_sapiens | Saudi_Arabia | 2015 | type5 |  |
| 540362575 | kf600612 | 4 | Homo_sapiens | Saudi_Arabia | 5-Feb-13 |  |  |
| 540362612 | kf600620 | 4 | Homo_sapiens | Saudi_Arabia | 7-May-13 |  |  |
| 540362667 | kf600628 | 3 | Homo_sapiens | Saudi_Arabia | 13-May-13 |  |  |
| 567322243 | kf961221 | 3 | Homo_sapiens | Qatar | 17-Oct-13 |  |  |
| 567322254 | kf961222 | 3 | Homo_sapiens | Saudi_Arabia | 8-Nov-13 |  |  |
| 582986757 | kj156934 | 3 | Homo_sapiens | Saudi_Arabia | 17-Jul-13 |  |  |
| 582986821 | kf156874 | 3 | Homo_sapiens | Saudi_Arabia | 5-Aug-13 | type3 |  |
| 582986845 | kf156910 | 3 | Homo_sapiens | Saudi_Arabia | 2-Jul-13 | type3 |  |
| 633896549 | kj813439 | 3 | Homo_sapiens | USA | 10-May-14 |  | 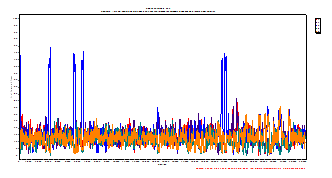 |
| 674305022 | km027262 | 3 | Homo_sapiens | United_Arab_Emirates | 7-Apr-14 |  | 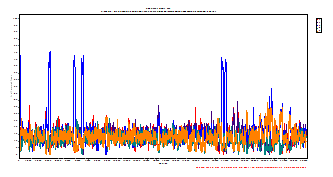 |
| 828177937 | KT006149 | 3 | Homo_sapiens | China | 2015 | type2 |  |
| 828436854 | KT026453 | 3 | Homo_sapiens | Saudi_Arabia | 2015 | type2 |  |
| 828436865 | KT026454 | 3 | Homo_sapiens | Saudi_Arabia | 2015 | type2 |  |
| 828436876 | KT026455 | 3 | Homo_sapiens | Saudi_Arabia | 2015 | type2 |  |
| 828436887 | KT026456 | 3 | Homo_sapiens | Saudi_Arabia | 2015 | type2 |  |
| 829021049 | KT029139 | 3 | Homo_sapiens | South Korea | 2015 | type2 |  |
| 511261278 | kf186564 | 2 | Homo_sapiens | Saudi_Arabia | 21-Apr-13 |  |  |
| 511261290 | kf186566 | 2 | Homo_sapiens | Saudi_Arabia | 9-May-13 |  |  |
| 511261302 | kf186567 | 2 | Homo_sapiens | Saudi_Arabia | 22-Apr-13 |  |  |
| 511261314 | kf186565 | 2 | Homo_sapiens | United_Arab_Emirates | 2013 |  |  |
| 540362655 | kf600627 | 2 | Homo_sapiens | Saudi_Arabia | 4-Jun-13 |  |  |
| 540362696 | kf600632 | 2 | Homo_sapiens | Saudi_Arabia | 30-May-13 |  |  |
| 540362710 | kf600634 | 2 | Homo_sapiens | Saudi_Arabia | 12-May-13 |  |  |
| 540362763 | kf600644 | 2 | Homo_sapiens | Saudi_Arabia | 11-May-13 |  |  |
| 540362775 | kf600645 | 2 | Homo_sapiens | Saudi_Arabia | 15-May-13 |  |  |
| 540362789 | kf600647 | 2 | Homo_sapiens | Saudi_Arabia | 23-May-13 |  |  |
| 540362808 | kf600651 | 2 | Homo_sapiens | Saudi_Arabia | 30-Oct-12 |  |  |
| 540362820 | kf600652 | 2 | Homo_sapiens | France | 7-May-13 |  |  |
| 582986797 | kj156866 | 2 | Homo_sapiens | Saudi_Arabia | 28-Aug-13 |  |  |
| 582986809 | kj156869 | 2 | Homo_sapiens | Saudi_Arabia | 12-Jun-13 |  |  |
| 657204369 | km015348 | 2 | Homo_sapiens | Saudi_Arabia | 13-Jun-12 | type1 |  |
| 746873493 | km210277 | 2 | Homo_sapiens | United_Kingdom | 10-Feb-13 | type1 |  |
| 746873505 | km210278 | 2 | Homo_sapiens | United_Arab_Emirates | 14-Jun | type1 |  |
| 540362681 | kf600630 | 1 | Homo_sapiens | Saudi_Arabia | 23-May-13 |  |  |
| 562738361 | kf745068 | 1 | Homo_sapiens | Qatar | 13-Oct-13 |  | 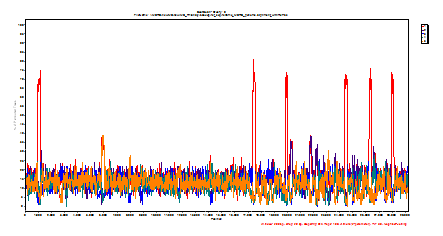 |
| 612348148 | kj650295 | 1 | Camelus_dromedarius | Saudi_Arabia | 30-Dec-13 |  |  |
| 612348159 | kj650296 | 1 | Camelus_dromedarius | Saudi_Arabia | 30-Nov-13 |  |  |
| 612348170 | kj650297 | 1 | Camelus_dromedarius | Qatar | 16-Feb-14 |  |  |
| 620988565 | kj713299 | 1 | Camelus_dromedarius | USA | 30-Apr-14 |  |  |
| 727377765 | kp209306 | 1 | Homo_sapiens | United_Arab_Emirates | 10-Apr-14 |  |  |
| 727377778 | kp209307 | 1 | Homo_sapiens | United_Arab_Emirates | 10-Apr-14 |  |  |
| 727377791 | kp209308 | 1 | Homo_sapiens | United_Arab_Emirates | 19-Apr-14 |  |  |
| 727377804 | kp209309 | 1 | Homo_sapiens | United_Arab_Emirates | 7-Mar-14 |  |  |
| 727377817 | kp209310 | 1 | Homo_sapiens | United_Arab_Emirates | 17-Apr-14 |  |  |
| 727377830 | kp209311 | 1 | Homo_sapiens | United_Arab_Emirates | 15-Nov-13 |  |  |
| 727377843 | kp209312 | 1 | Homo_sapiens | United_Arab_Emirates | 13-Apr-14 |  |  |
| 727377856 | kp209313 | 1 | Homo_sapiens | USA | 14-Jun |  |  |
| 732559229 | kj361500 | 1 | Homo_sapiens | France | 7-May-13 |  |  |
| 732559241 | kj361501 | 1 | Homo_sapiens | France | 7-May-13 |  |  |
| 732559253 | kj361502 | 1 | Homo_sapiens | France | 7-May-13 |  |  |
| 732559265 | kj361503 | 1 | Homo_sapiens | United_Kingdom | 13-Feb-13 |  |  |
| 752855041 | kp719927 | 1 | Camelus_dromedarius | United_Arab_Emirates | 14-Jun |  |  |
| 752855053 | kp719928 | 1 | Camelus_dromedarius | United_Arab_Emirates | 14-Jun |  |  |
| 752855065 | kp719928 | 1 | Camelus_dromedarius | United_Arab_Emirates | 14-Jun |  |  |
| 752855077 | kp719929 | 1 | Camelus_dromedarius | United_Arab_Emirates | 14-Jun |  |  |
| 752855089 | kp719931 | 1 | Camelus_dromedarius | United_Arab_Emirates | 14-Jun |  |  |
| 752855101 | kp719932 | 1 | Camelus_dromedarius | United_Arab_Emirates | 2014 |  |  |
| 752855113 | kp719933 | 1 | Camelus_dromedarius | Saudi_Arabia | 21-Jan-15 |  |  |

Perl script:

#! /usr/bin/perl

use strict;

print "Usage: perl random.pl INPUTFILE SAMPLESIZE\nINPUTFILE should be fasta format";

my $input = shift;

my $samplesize = shift;

my $out = $input.".$samplesize.out.fas";

my $count = 0;

my %seq;

open (IN,"$input")||die $!;

open (OUT,">$out");

$/=">";

while (my $item = <IN>){

chomp $item;

if (length($item) >100){

$count++;

$seq{$count}=$item;

}

}

#############sampling with replacement################

for (my $i = 1;$i<=$samplesize;$i++){

my $sample=int(rand($count));

print OUT ">$seq{$sample}";

}

#############sampling without peplacement#############

my @resample;

my %resample;

my $samplenum=0;

for (my $i = 1;$i<=3*$count;$i++){

my $sample=int(rand($count));

if (exists $seq{$sample} && !exists $resample{$sample} && $samplenum<$samplesize){

$resample{$sample}=$seq{$sample};

push @resample, $sample;

$samplenum++;

}

else {

}

}

foreach my $resample(@resample){

print OUT ">$seq{$resample}";

}

#################################################

close IN;

close OUT;

exit;
